# Alcohols Effect on Critic Micelle Concentration of Polysorbate 20 and Cetyl Trimethyl Ammonium Bromine Mixed Solutions

**DOI:** 10.1007/s11743-012-1429-x

**Published:** 2013-01-05

**Authors:** Taliha Sidim, Gökhan Acar

**Affiliations:** Department of Chemistry, Faculty of Science, Trakya University, Edirne, Turkey

**Keywords:** Surface tension, Conductivity, Critical micelle concentration, Nonionic surfactants

## Abstract

In this research, the micellar behavior of a cationic surfactant, cetyl trimethyl ammonium bromide (CTAB) and an nonionic surfactant, polysorbate 20 (Polyoxyethylene (20) sorbitan monolaurate) in different alcohol solutions media was investigated over the temperature range 293.15–313.15 K. The interaction between two surfactants in binary systems can be determined by calculating the values of their β parameters. The critical micelle concentrations (CMC) of the micelles were determined from the surface tension, the conductivity at different temperatures. The CMC behavior of CTAB and polysorbate 20 was analyzed in terms of the effect of temperature and the increase in the alcohol carbon chain. Changes in the critical micelle concentration of mixed surfactant systems of different alcohol solutions were measured. The CMC decreased sharply as the hydrocarbon chain length of the alcohols becomes larger. This shows that the more hydrophobic alcohols are, the more marked a decrease in CMC is observed.

## Introduction

The widespread industrial, technological, and domestic applications of surfactants usually involve mixtures. This is often because the materials that are used are impure, but more importantly because different surfactants are often deliberately mixed to provide enhanced performance [1].

It is necessary in most practical applications to choose mixtures of surfactants to conveniently tune the desired properties of the formulation. Mixtures of surface active materials often show synergistic interactions which would be manifested as enhanced surface activity, spreading, wetting, foaming, detergency, and many other phenomena. Mixtures of nonionic surfactants tend to behave ideally while ionic surfactants can exhibit departure from ideality [2].

From a fundamental point of view, the mixtures of ionic–nonionic surfactants are more interesting because they often exhibit a highly nonideal behavior. Adding a nonionic surfactant into an ionic surfactant micelle can reduce the electrostatic repulsions between the charged surfactant heads and greatly facilitate mixed micelle formation. In the literature, it is possible to find data concerning the anionic/nonionic mixtures of two surfactants rather than those of cationic/nonionic ones which are also used in many processes such as detergents for some materials [3].

The concentration of surfactant needed to initiate micelle formation is called the critical micelle concentration (CMC). The value of the CMC can be determined by the change in the physicochemical properties of the surfactant solution as the concentration of the amphiphile is increased [4–7]. Some of the physical properties that have been studied for this purpose include solution detergency, viscosity, density, conductivity, surface tension, osmotic pressure, interfacial tension, refractive index and light scattering.

Surfactants are mostly low-molecular weight compounds, so when dissolved, they form true solutions in concentration ranges below the CMC. Micelles are aggregates of a large number of simple molecules or ions of surfactants (e.g. several dozens), so the resulting size of such structures is in the colloidal range. For this reason the micelle solutions of surfactants are regarded as association colloids. It is essential to employ physical methodologies which are highly sensitive to structural changes for determining the CMC. The existence of CMC indicates aggregation of amphiphilic molecules in solutions. The knowledge of the CMC is important for the calculation of the thermodynamic parameters, which confirms the scientific interest of a precise determination of the CMC [8]. The CMC in aqueous solution is influenced by the degree of binding of counter ions to the micelles. For aqueous systems, the increased binding of the counter ion to the surfactant causes a decrease in the CMC and an increase in the aggregation number [9].

Since water alcohol-surfactant systems are frequently used as media in the studies of chemical equilibria and reaction rates, it is essential to investigate the effect of the nature of the alkyl groups in the alcohol on the CMC of the surfactants. Addition of alcohols to aqueous solutions of surfactants has allowed the investigation to be made of the effect of hydrophobic interactions on the micellar structure [10].

The effect of the presence of additives on the CMC of surfactants has been widely studied. It is generally accepted that the alcohol binds to the micelle in the surface region, leading to three principal effects: (a) The alcohol molecules intercalate between the surfactants ionic head groups to decrease the micelle surface area per head group and increase the ionization. This effect is correlated with modification of the growth and shape of the micelle. It seems to be a function of the mole fraction of alcohol at the micellar interface but is independent of the type of alcohol. (b) The dielectric constant at the micellar interface decreases probably due to the replacement of water molecules in the interface region by alcohol molecules. (c) The molecular order of the interface region of the micelle changes [11–16].

The effect of alcohol addition in micellar solutions of various surfactants in aqueous solutions has not been studied on a large scale. The purpose of this work was to study the effects of some long-chain alcohols on the micellization process of sodium dodecyl sulfate (SDS) and sodium laurate (SLA) in *N*,*N* dimethyl formamide (DMF) solution. These surfactants were chosen because they are widely used and commercially available. Our work essentially involves the determination of the CMC by means of surface tension and conductivity measurements. In the presence of various chain alcohols, the relationships among the CMC, thermodynamic functions, and the alcohol carbon number and concentration in DMF during the micellization process are discussed.

## Experimental Procedures

### Materials

Polyoxyethylene (20) sorbitan monolaurate (Tween 20 is the commercial name) abbreviated as PS20 in what follows, and cetyl trimethyl ammonium bromide (CTAB) were used as received from Aldrich. Solutions including alcohol of individual surfactants and CTAB and PS20 mixtures at different ratios of CTAB to PS20 were prepared using doubly distilled and deionized water (GFL-2102). The surface tension of water was checked before the solution preparation. All the solutions were measured under the thermostated conditions at 293.15, 298.15, 303.15, 308.15 and 313.15 K with an accuracy of ±0.1 K.

### Procedures

#### Preparation of the Mixed Surfactant Solutions

Doubly distilled water from an all-glass apparatus was used to prepare all solutions. The surface tension of the water was 72.8 dyne cm^−1^ at 25 °C. Into several 100-mL beakers, aliquots of a given concentration of cationic surfactants (1 × 10^−2^ mol/L) were placed, followed by the addition of a given concentration of Tween 20 (1 × 10^−2^ mol/L). The mixtures were stirred and diluted stepwise with water. These mixtures were kept for 4 h under thermostatted conditions at different temperatures in order to establish equilibrium.

#### Surface Tension Measurements

The surface tensions of aqueous solutions of single and mixed surfactants at various concentrations were measured on the KSV SIGMA 702 ring tensiometer. The value of the surface tension was the average of the three separate measurements. All the measurements were taken at 298 K. The surface tension measurements were made at 298 K under atmospheric pressure by the ring method. The platinum ring was thoroughly cleansed, and flame dried before each measurement. The measurements were taken in such a way that the vertically hung ring was dipped into the liquid to measure its surface tension. It was then subsequently pulled out. The maximum force needed to pull the ring through the interface was then expressed as the surface tension, *γ* (mN/m). The measurements of the surface tension of pure water at 298 K were performed to calibrate the tensiometer and to check the cleanliness of the glassware. In all cases, more than 10 successive measurements were carried out, and the standard deviation did not exceed ±0.2 mN/m. The temperature was controlled within ±0.1 K.

#### Conductometric Measurements

The conductometric measurements were taken with a Jenway (UK) conductometer using a cell with a cell constant of 0.92 cm^−1^. Accuracy of the measured conductance was within 0.01 μS. The surfactant conductance was measured after thorough mixing and temperature equilibration. The break point in the plot of either the equivalent conductivity versus the square root of the total surfactant concentration or the molar conductivity versus the total surfactant concentration was taken as the CMC at the mole fraction.

#### CMC Determinations

The surface tension of aqueous solutions of single and mixed surfactants at various concentrations were determined using the Du Nouy ring method at constant temperature. The CMC values were determined by break points in the plot of surface tension values against the concentration values. The CMC values were found to be in agreement with the measured solution conductivity and density. The surface tension data show that the value of CMC of a single surfactant did not change within the temperature range of 298–323 K whereas the value of the CMC of mixed surfactants decreases as the temperature rises above 298 K. This is the reason of the selection of a wide concentration range.

The break point in the plot of either the equivalent conductivity versus the square root of the total surfactant concentration or the molar conductivity versus the total surfactant concentration was taken as the CMC at the mole fraction.

## Results and Discussion

The surface tensions and conductivities were measured as a function of surfactant concentration at 293.15, 298.15, 303.15, 308.15 and 313.15 K. The CMC values of different combinations of the binary (PS20/CTAB) mixtures in different alcohol solutions were determined from the surface tension and conductivity versus surfactant concentration plots, at different temperatures. CMC data are listed in Tables 1, 2, 3, 4 and 5 for different alcohol solutions at five different temperatures. The conclusions from conductivity measurements coincide with those of surface tension as shown Tables 1, 2, 3, 4, 5, 6, 7, 8, 9 and 10.

**Table 1:** Critical micellar concentration for the PS20/CTAB systems as a function of the mole fraction of the nonionic surfactant in different alcohol solutions at 20 °C

PS20/CTAB	10 % Methanol		10 % Ethanol		10 % Propanol		10 % Butanol	
	Surface tension	Conductivity	Surface tension	Conductivity	Surface tension	Conductivity	Surface tension	Conductivity
0	0.46	0.41	0.73	0.84	0.55	0.62	0.12	0.23
0.2	0.62	0.69	0.73	0.72	0.47	0.46	0.17	0.06
0.4	0.28	0.61	0.28	0.48	0.11	0.32	0.12	0.11
0.6	0.27	0.38	0.53	0.67	0.11	0.28	0.10	0.07
0.8	0.29	0.27	0.30	0.33	0.22	0.25	0.12	0.09
1	0.19	0.05	0.12	0.76	0.11	0.38	0.09	0.13

**Table 2:** Critical micellar concentration for the PS20/CTAB systems as a function of the mole fraction of the nonionic surfactant in different alcohol solutions at 25 °C

PS20/CTAB conductivity	10 % Methanol		10 % Ethanol		10 % Propanol		10 % Butanol	
	Surface tension	Conductivity	Surface tension	Conductivity	Surface tension	Conductivity	Surface tension	Conductivity
0	0.54	0.40	0.43	0.37	0.27	0.25	0.20	0.22
0.2	0.60	0.57	0.51	0.49	0.47	0.44	0.15	0.07
0.4	0.11	0.13	0.33	0.49	0.27	0.33	0.13	0.07
0.6	0.24	0.11	0.13	0.39	0.15	0.10	0.12	0.08
0.8	0.29	0.10	0.15	0.07	0.11	0.06	0.10	0.08
1.0	0.11	0.17	0.52	0.46	0.51	0.49	0.17	0.25

**Table 3:** Critical micellar concentration for the PS20/CTAB systems as a function of the mole fraction of the nonionic surfactant in different alcohol solutions at 30 °C

PS20/CTAB	10 % Methanol		10 % Ethanol		10 % Propanol		10 % Butanol	
	Surface tension	Conductivity	Surface tension	Conductivity	Surface tension	Conductivity	Surface tension	Conductivity
0	0.50	0.54	0.44	0.40	0.50	0.65	0.13	0.17
0.2	0.49	0.45	0.44	0.43	0.13	0.40	0.09	0.07
0.4	0.13	0.27	0.17	0.10	0.23	0.38	0.10	0.10
0.6	0.11	0.31	0.44	0.41	0.47	0.50	0.12	0.11
0.8	0.09	0.07	0.13	0.09	0.11	0.05	0.10	0.08
1.0	0.11	0.05	0.12	0.08	0.10	0.08	0.09	0.08

**Table 4:** Critical micellar concentration for the PS20/CTAB systems as a function of the mole fraction of the nonionic surfactant in different alcohol solutions at 35 °C

PS20/CTAB	10 % Methanol		10 % Ethanol		10 % Propanol		10 % Butanol	
	Surface tension	Conductivity	Surface tension	Conductivity	Surface tension	Conductivity	Surface tension	Conductivity
0	0.50	0.54	0.41	0.39	0.44	0.54	0.26	0.19
0.2	0.51	0.45	0.47	0.47	0.47	0.49	0.12	0.07
0.4	0.13	0.49	0.53	0.30	0.11	0.29	0.11	0.10
0.6	0.11	0.06	0.47	0.41	0.45	0.47	0.12	0.10
0.8	0.09	0.03	0.13	0.37	0.20	0.05	0.11	0.10
1	0.17	0.10	0.42	0.38	0.32	0.37	0.23	0.36

**Table 5:** Critical micellar concentration for the PS20/CTAB systems as a function of the mole fraction of the nonionic surfactant in different alcohol solutions at 40 °C

PS20/CTAB	10 % Methanol		10 % Ethanol		10 % Propanol		10 % Butanol	
	Surface tension	Conductivity	Surface tension	Conductivity	Surface tension	Conductivity	Surface tension	Conductivity
0	0.46	0.58	0.43	0.45	0.35	0.49	0.12	0.15
0.2	0.55	0.45	0.46	0.47	0.47	0.45	0.10	0.11
0.4	0.26	0.20	0.68	0.68	0.45	0.45	0.10	0.10
0.6	0.11	0.11	0.47	0.47	0.47	0.47	0.10	0.11
0.8	0.26	0.28	0.45	0.46	0.45	0.40	0.13	0.12
1.0	0.19	0.10	0.12	0.10	0.11	0.10	0.09	0.09

**Table 6:** Minimum surface tension values for the PS20/CTAB systems as a function of the mole fraction of the nonionic surfactant in different alcohol solutions at 20 °C

PS20/CTAB	10 % Methanol	10 % Ethanol	10 % Propanol	10 % Butanol
	*γ* min (mN/m)	*γ* min (mN/m)	*γ* min (mN/m)	*γ* min (mN/m)
0	40.47	35.22	34.87	25.52
0.2	37.62	35.6	35.67	25.07
0.4	37.62	38.23	40.14	24.93
0.6	36.32	36.97	42.95	25.51
0.8	37.62	36.97	38.78	26.21
1	33.10	36.97	34.87	25.92

**Table 7:** Minimum surface tension values for the PS20/CTAB systems as a function of the mole fraction of the nonionic surfactant in different alcohol solutions at 25 °C

PS20/CTAB	10 % Methanol	10 % Ethanol	10 % Propanol	10 % Butanol
	*γ* min	*γ* min	*γ* min	*γ* min
0	40.45	39.99	33.93	24.51
0.2	33.21	36.45	35.25	24.33
0.4	33.96	35.29	34.71	24.63
0.6	34.67	37.28	34.76	24.72
0.8	34.57	37.21	34.17	25.13
1	32.2	37.24	33.49	24.63

**Table 8:** Minimum surface tension values for the PS20/CTAB systems as a function of the mole fraction of the nonionic surfactant in different alcohol solutions at 30 °C

PS20/CTAB	10 % Methanol	10 % Ethanol	10 % Propanol	10 % Butanol
	*γ* min (mN/m)	*γ* min (mN/m)	*γ* min (mN/m)	*γ* min (mN/m)
0	40.05	36.74	35.51	25.90
0.2	33.46	36.78	33.03	25.56
0.4	35.27	36.73	34.21	43.38
0.6	35.74	36.26	32.41	25.91
0.8	35.79	37.78	33.62	44.43
1	33.87	34.45	30.99	39.9

**Table 9:** Minimum surface tension values for the PS20/CTAB systems as a function of the mole fraction of the nonionic surfactant in different alcohol solutions at 35 °C

PS20/CTAB	10 % Methanol	10 % Ethanol	10 % Propanol	10 % Butanol
	*γ* min (mN/m)	*γ* min (mN/m)	*γ* min (mN/m)	*γ* min (mN/m)
0	36.83	36.40	31.47	25.66
0.2	31.05	35.82	32.69	24.60
0.4	34.32	35.41	34.00	25.02
0.6	35.91	35.90	31.77	25.40
0.8	34.74	36.40	33.21	26.22
1	32.97	34.39	31.50	25.57

**Table 10:** Minimum surface tension values for the PS20/CTAB systems as a function of the mole fraction of the nonionic surfactant in different alcohol solutions at 40 °C

PS20/CTAB	10 % Methanol	10 % Ethanol	10 % Propanol	10 % Butanol
	*γ* min (mN/m)	*γ* min (mN/m)	*γ* min (mN/m)	*γ* min (mN/m)
0	34.24	35.58	33.17	23.78
0.2	31.99	34.30	32.16	23.48
0.4	33.98	34.41	33.48	23.56
0.6	34.24	35.47	31.48	23.65
0.8	33.20	36.17	32.84	24.17
1	32.17	33.50	30.35	24.26

The CMC value of the mixture decreases after the initial addition of PS 20 (nonionic surfactant) indicating slight CMC synergism (i.e. the CMC of the mixture is lower than the CMC of its individual components). The critical micelle concentrations of mixed surfactants are lower than that of sole CTAB and very close to that of pure PS20. In this respect, this experimental results for fresh solutions agrees with those observed by Mata, who found a decrease in the CMC of mixtures with increase in the mole fraction of PS20, however, the CMC of the mixed system at any composition could not be reduced to be lower than that of pure PS20. A similar decrease in CMC was also observed in the mixture of dimeric anionic and nonionic surfactants [17–19].

The hydrophobic effect associated with the hydrophobic moiety of alcohol molecules also favors micellization and increases as the length of the hydrocarbon chain of the alcohol series increases. This explains the increased lowering of the CMC as the number of carbon atoms increases in alcohol series [20–22].

The changes in CMC, with increasing addition of methanol, ethanol, *n*-propanol, and butanol are reported in Tables 1, 2, 3, 4 and 5. The reason for the CMC decreasing is that the major factor that determines the intermicellar solubility of long chain alcohols is the change in the hydrophilic balance of the micelle during the inclusion of alcohol in it [23, 24].

**Fig. 1:**
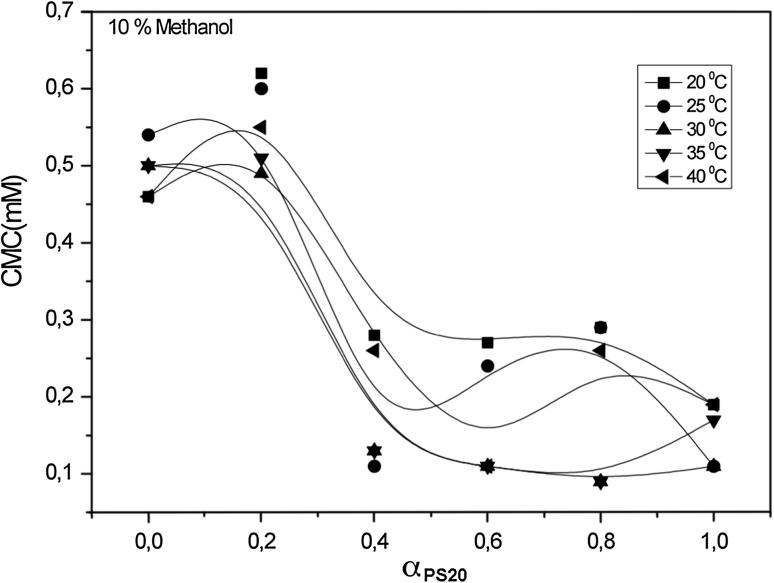
Variation of critical micelle concentration versus mole fraction of ionic surfactant for mixed surfactant system in methanol at 20, 25, 30, 35 and 40 °C

**Fig. 2:**
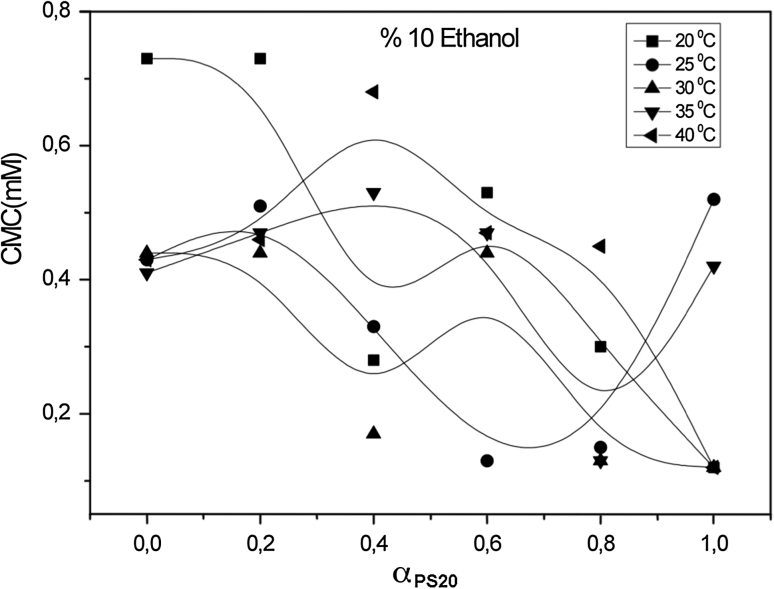
Variation of critical micelle concentration versus mole fraction of ionic surfactant for mixed surfactant system in ethanol at 20, 25, 30, 35 and 40 °C

**Fig. 3:**
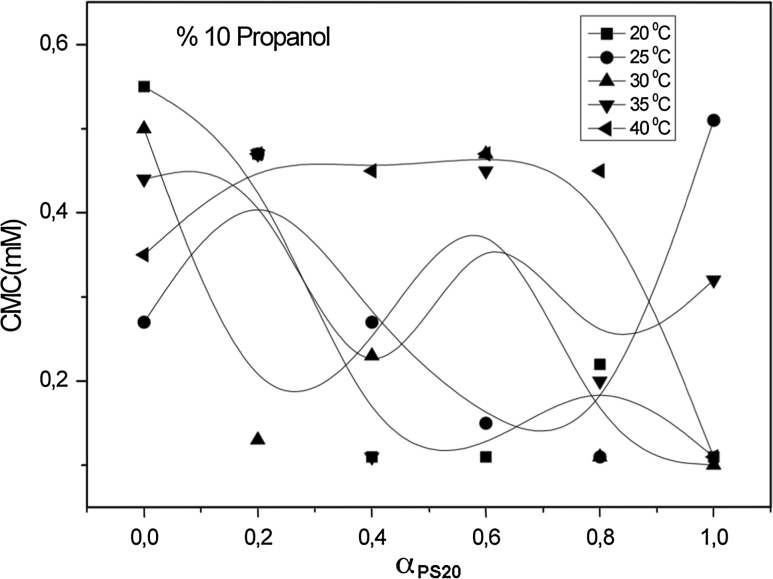
Variation of critical micelle concentration versus mole fraction of ionic surfactant for mixed surfactant system in propanol at 20, 25, 30, 35 and 40 °C

**Fig. 4:**
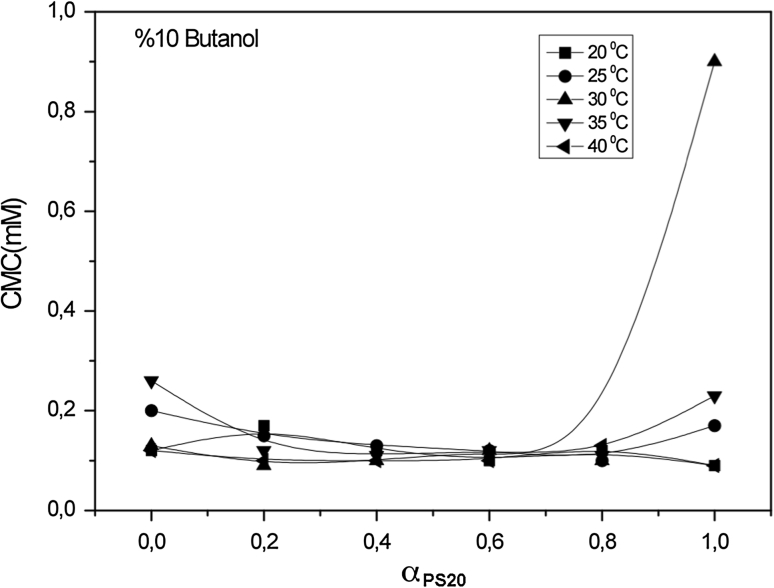
Variation of critical micelle concentration versus mole fraction of ionic surfactant for mixed surfactant system in butanol at 20, 25, 30, 35 and 40 °C

For systems containing an identical alcohol concentration at the same temperature, the CMC values decrease as the alcohol carbon number increases. The CMC values decrease as the number of carbon atoms in the hydrophobic group increases. For a given class, the CMC values decrease as the number of carbon atoms in the hydrophobic fragment increases. As shown in Figs. 1, 2, 3 and 4, values of the CMC increase with increasing temperature at constant concentration for each alcohol, but decrease with increasing carbon number of the alcohol at constant temperature and alcohol concentration. Our results in the case of binary mixtures have corroborated these findings [25–27].

The CMC values of nonionic surfactants depend on the length of both the lipophilic and hydrophilic parts of their molecules. The CMC decreases with increasing length of the hydrophobic moiety for a fixed hydrophilic group. The CMC of nonionic surfactant decreases with decreasing polyoxyethylene content in the molecule. Due to different structural consequences of intermolecular interactions caused by the different chain-lengths of the alcohols the CMC decreases sharply as the hydrocarbon chain length of alcohols becomes larger. Critical micelle concentration decreases as the nonionic surfactant mole fraction number increases. It has been reported that the synergism of solubilization in mixed micelle solutions decreased with increases in the mole fraction of the nonionic surfactant [28].

The interaction between two surfactants in binary systems can be determined by calculating the values of their β parameters as is shown in Table 11. Since the value of the β parameter is proportional to the free energy of mixing of the system, a negative β value indicates that the attractive interaction between the two different surfactants is stronger than the attractive interaction between each type of surfactant and another molecule of the same type or that the repulsive interaction between the two different surfactants is weaker than the self-repulsion between two individual surfactants of the same type.

**Table 11:** Micellar composition (*x* ^*M*^) and interaction parameters *β* ^*σ*^ and *β* ^*M*^ values in different stoichiometric compositions for mixed systems of CTAB/PS20

*β* ^*M*^	*α* (mole fraction of nonionic surfactant)	*x* ^*M*^ (micellar composition)	*β* ^*σ*^
0.2	0.67	−2.80	−14.78
0.4	0.75	−9.71	−15.36
0.6	0.78	−5.42	−13.64
0.8	0.89	9.38	−132.50

## Conclusions

In this research, the micellar behavior of cationic surfactant, cetyl trimethyl ammonium bromide and nonionic surfactant, polyoxyethylene (20) sorbitan monolaurate in different alcohol solutions media was investigated with the help of surface tension and conductivity over the temperature range 293.15–323.15 K. The CMC and of the micelles were determined from the surface tension and the conductivity measurements at different temperatures. The CMC decreased to a certain minimum and then increased with the temperature, displaying a U-shaped behavior. It was observed that by changing the counter ion from methanol to propanol along with the increase in carbon chain, the CMC shows a decrease. In an alcohol series, the hydrophobic character increases as the number of hydroxyl groups increases. The CMC decreases sharply as the hydrocarbon chain length of alcohols becomes larger. It shows that the more hydrophobic alcohols are, the more marked a decrease in CMC is observed.
